# An *Arthrobacter citreus* strain suitable for degrading ε-caprolactam in polyamide waste and accumulation of glutamic acid

**DOI:** 10.1186/s13568-019-0887-1

**Published:** 2019-10-11

**Authors:** Nandita N. Baxi, Shweta Patel, Dipeksha Hansoti

**Affiliations:** Department of Microbiology and Biotechnology Centre, Faculty of Science, Maharaja Sayajirao University of Baroda, Vadodara, 390002 India

**Keywords:** *Arthrobacter citreus*, 6-Aminocaproic acid, ε-Caprolactam, Glutamic acid, Polyamide waste

## Abstract

ε-Caprolactam-a toxic xenobiotic compound present in industrial polyamide waste was found to be degraded by caprolactam-degrading bacteria. *Arthrobacter citreus* was able to utilize up to 20 g ε-caprolactam/l as the sole source of carbon more efficiently as compared to the other Gram positive caprolactam-degrading bacteria *Rhodococcus rhodochrous* and *Bacillus sphaericus*. The cells of *A*. *citreus* remained viable in medium up to 40 g caprolactam/l. The degradation of 10 g caprolactam/l by *A. citreus*, when supplied as the sole source of carbon and nitrogen lead to the formation of 6-aminocaproic acid which was detected in broth and there was also an increase in the ammonium content. One of the other metabolites found to consistently accumulate in extracellular medium during the utilization of caprolactam by *A. citreus* was glutamic acid, though not reported in case of other caprolactam-degrading bacteria. *A. citreus* could metabolise caprolactam to form non toxic products such as 6-aminocaproic acid and glutamic acid which are amino acids of physiological and commercial importance. In the presence of 6-aminocaproic acid, the rate of caprolactam utilization by *A. citreus* was decreased but not inhibited and the viable count of cells was found to increase using both the substrates simultaneously. *A. citreus* was also suitable for degradation of caprolactam in presence of low phosphate as prevalent in soil, and in sterile soil without the supplementation of any other carbon or nitrogen, as well as in native non sterile soil where other microorganisms are present.

## Introduction

ε-Caprolactam is a man-made compound used as the monomer to produce the polymer nylon-6. Caprolactam is thus industrially important and is typically called as a high volume compound due to its large scale worldwide production and its consumption in nylon-6 industries (Linde and Fisher 2004). As a consequence of its use as a raw material in industries, caprolactam is also found to be present in solid wastes and wastewaters of industrial units which manufacture either nylon-6 or caprolactam. It is not surprising that caprolactam unintentionally enters the environment and is detected in soil and water and even detected within plants and causes adverse effects (Kalinova et al. 2014). Several toxic effects of caprolactam to various living forms are reported (Shama and Wase 1981; Goldbatt et al. 1954). In the environment the capacity to degrade a large number of xenobiotic compounds is attributed to diverse microbial activity. During aerobic microbial degradation, xenobiotic compounds maybe converted to non toxic products but this is at the cost of depletion of oxygen from the polluted environment and this is a pollutional load on the environment. However microorganisms of only a few genera are reported to utilize and degrade caprolactam (Shama and Wase 1981; Wang and Lee 2007; Sanuth et al. 2013) and thus it may persist in environment for a varying and indefinite time period.

The main product obtained when bacteria degrade caprolactam is the corresponding linear compound: 6-aminocaproic acid which is converted by further metabolism to adipic acid and succinic acid. The metabolic products obtained during the degradation of caprolactam by bacteria, the enzymes involved and the genes responsible for coding for these enzymes have been recently studied in *Pseudomonas jessenii* (Otzen et al. 2018). In some other caprolactam-degrading *Pseudomonas* strains the role of plasmids has been reported (Grishchenkov et al. 1993). Although caprolactam degradation has been studied by various workers using different bacteria either in media only or for effluent treatment, the degradation of caprolactam in oligomer waste and the in situ bioremediation of soil polluted with caprolactam has not been reported. Biodegradation in soil requires strains to be successful in bringing about degradation in the complex physico-chemical soil condition where other microflora is also present. In the present study we report a strain: *Arthrobacter citreus* suitable for biodegradation of caprolactam in media as the sole source of carbon and nitrogen in absence of additional nutrients and growth factors and even in soil where the level of nutrients is low. In earlier reports of caprolactam-degrading bacteria such suitable properties have not been described and there are no reports of degradation of caprolactam from oligomer waste. Further, during the utilization of caprolactam by *A. citreus*, along with 6-aminocaproic acid, glutamic acid was obtained as a major extracellular product. In wild type strains of bacteria, such metabolites are excreted into medium only when the concentration within the cell is in excess compared to that required for growth of cells. There are no reports so far of caprolactam-degrading bacteria found to accumulate glutamic acid extracellularly. The present study thus shows the capability of *A. citreus* cells to degrade caprolactam in situ and also its conversion to amino acids which can be easily assimilated further or accumulated under appropriate conditions.

## Materials and methods

### Bacterial cultures and media

The caprolactam-degrading bacteria were previously isolated from industrial soil and identified at Microbial Type Culture Collection, MTCC, Institute of Microbial Technology, IMTECH, Chandigarh, India. The cultures are *A. citreus* (GENBANK sequence Accession number JX129362), *Rhodococcus rhodochrous*, *B. sphaericus* and *Alcaligenes faecalis.* The MTCC, IMTECH Accession numbers of deposited cultures—*R. rhodochrous* and *A. citreus* are 3096 and 3097 respectively and the other cultures were maintained in department laboratory culture collection. For routine maintenance and cultivation of the bacteria, synthetic medium (composition in g/l: KH_2_PO_4_ 0.2, K_2_HPO_4_ 0.6, NaCl 0.3, MgSO_4_·7H_2_O 0.2, CaCl_2_·2H_2_O 0.1, FeCl_3_ 0.1, pH 7.2) containing caprolactam as the sole source of carbon and nitrogen (Baxi and Shah 2002) was used unless otherwise mentioned. For inoculum development the bacterial cultures were transferred from agar slants into tubes with 5.0 ml synthetic medium containing caprolactam and incubated on a rotary shaker (180 rpm) for 24 h at 30 (± 2) °C. The absorbance of cultures at 600 nm when adjusted to 1 gave approximately a viable count of 10^8^ cfu/ml and was subsequently used as an inoculum (1%) for shake flask studies. After inoculation of medium the initial OD_600_ was 0.2 to 0.3

### Biodegradation studies

In order to study the biodegradation of the caprolactam in the polyamide waste which was in the form of a soft solid, the oligomers in the waste were first solubilised by heating at 80 °C at concentration of 1 g/100 ml of synthetic basal medium. If solubilisation was done using more than 1 g/100 ml of waste, the solubilisation was not complete. Thus such medium contained monomer caprolactam and other oligomer constituents of waste as the sources of carbon and nitrogen for the inoculated bacteria. Caprolactam present in the oligomer waste medium used for biodegradation study was estimated as 0.32%. For shake flask studies of biodegradation, 50 ml of such medium in 250 ml Erlenmeyer flasks, was inoculated with mixture of caprolactam-degrading bacterial isolates and incubated on a rotary shaker (180 rpm) at 30 (± 2) °C for appropriate incubation time. Biodegradation of only caprolactam was studied using the same experimental set up but with basal synthetic medium with caprolactam as substrate. In medium in the shake flask experiments caprolactam was supplied initially at 0.5, 1, 1.5% as sole source of carbon. Subsequently 1% w/v caprolactam was supplied as sole carbon and nitrogen source. In two substrate experiments caprolactam along with another substrate 6-ACA or sodium citrate was used. For biodegradation in soil, caprolactam (0.2% w/w) was added to fertile garden soil and inoculated with *A. citreus*.

For all the biodegradation experiments, medium samples or soil extracts were withdrawn at appropriate time intervals, centrifuged at 10,000×*g* (Heraeus microcentrifuge) for 20 min and the cell free supernatant was used for checking the degradation of caprolactam and other compounds by TLC, HPTLC or spectrophotometric estimation. Growth of bacteria was measured by monitoring either viable count (colony forming units/ml) or the absorbance of the culture broth at 600 nm. Average values of three experiments and estimations are reported.

### Estimations

Caprolactam was estimated using a spectrophotometric method (Bergmann 1952). 1.0 ml aliquots of standard caprolactam (up to 10 mM) or culture supernatants containing residual caprolactam were mixed with 2.0 ml of alkaline hydroxylamine reagent (equal volumes of 2.0 N hydroxylamine sulphate and 3.5 N sodium hydroxide), covered and heated for 7 h in a water bath at 60 °C. After cooling the reaction mixture to ambient temperature, 1.0 ml of 3.5 N hydrochloric acid and 1.0 ml of 0.74 M ferric chloride in 0.1 N hydrochloric acid solution were added and the absorbance was measured at 540 nm.

6-Aminocaproic acid was estimated using a quantitative ninhydrin reagent based spectrophotometric method. 2.0 ml aliquots of standard 6-ACA (0.05 to 0.2 mM) or culture supernatants (priorly selected by TLC as those having only 6-ACA) were mixed with 2.0 ml of buffered ninhydrin-reagent [2% (w/v) ninhydrin and 3% (w/v) hydrindantin in 3:1 mixture of methyl cellosolve and 4 N sodium acetate buffer having pH 5.5], covered with marble and heated for exactly 15 min at 100 °C in a water bath after which the reaction mixture was diluted with 3.0 ml of 50% ethanol, cooled to room temperature and the absorbance was read at 570 nm.

Glutamic acid was estimated using the spectrophotometric method (Zamir and Lichtenstein 1955) wherein glutamic acid is *selectively* converted to pyrrolidone carboxylic acid (PCA) under conditions which do not affect other amino acids and this method is thus used to *specifically detect and estimate* glutamic acid present in hydrolysed protein samples where all other amino acids are also present. Either sodium glutamate (1–20 mg/ml) or culture supernatant containing glutamic acid, was adjusted to pH 3–4 by addition of 1 N HCl, autoclaved for 4 h at 121 °C, 15 psi. Aliquotes of PCA formed (equivalent to 1 to 20 mg glutamic acid) were taken in a 1.0 ml system and mixed with 0.5 ml of a 50% (w/v) solution of hydroxylamine hydrochloride in 2 N NaOH and heated for 15 min in a water bath at 100 °C to form hydroxamic acid. After cooling to ambient temperature, 2.5 ml of the ferric chloride reagent (a mixture of equal volumes of 2.5 N HCl, a 15% solution of trichloroacetic acid, and a 10% solution of ferric chloride in 0.1 N HCl) was added. The absorbance of stable product was measured at 540 nm after 2 min as per standardization in the present study.

Ammonium nitrogen was estimated spectrophotometrically as described by Bergersen (1980). 1.0 ml aliquots of either 15 to 150 μg ammonium N/ml in form of ammonium salt or culture supernatant were mixed together with 1.0 ml phenol–nitroprusside reagent [5% (v/v) phenol with 0.025% (w/v) sodium nitroprusside] and 1.0 ml alkaline sodium hypochlorite reagent [2.5% (w/v) NaOH with 0.21% (v/v) sodium hypochlorite] and after incubation for 30 min at ambient temperature the absorbance was read at 625 nm.

### Thin layer chromatography (TLC) and HPTLC

Caprolactam, oligomers, 6 aminocaproic acid, glutamic acid were resolved using TLC. For HPTLC, 3 μl samples were applied on tracks on 20 cm × 10 cm TLC plates; 0.2 mm thick precoated with silica gel G F24 (E. Merck, India) as the adsorbent, using Inomat IV applicator in form of 9 mm band length. Plates were placed in trough for run up to 90 mm, in appropriate solvent system (as in figure legends). The resolved components were detected by spraying with Dragendorff’s reagent for detection of cyclic compounds with lactam group and Ninhydrin reagent for linear oligomers and amino acids. The developed HPTLC plates were scanned at 570 nm (D2 lamp, slit dimension 4 × 0.3 mm) for 2D spectra to match standard and unknown spots using CAMAG automatic scanner ATS4-140608 S/N and with win CATS software1.02.13.

## Results

### Biodegradation of caprolactam in polyamide oligomer waste

**Fig. 1:**
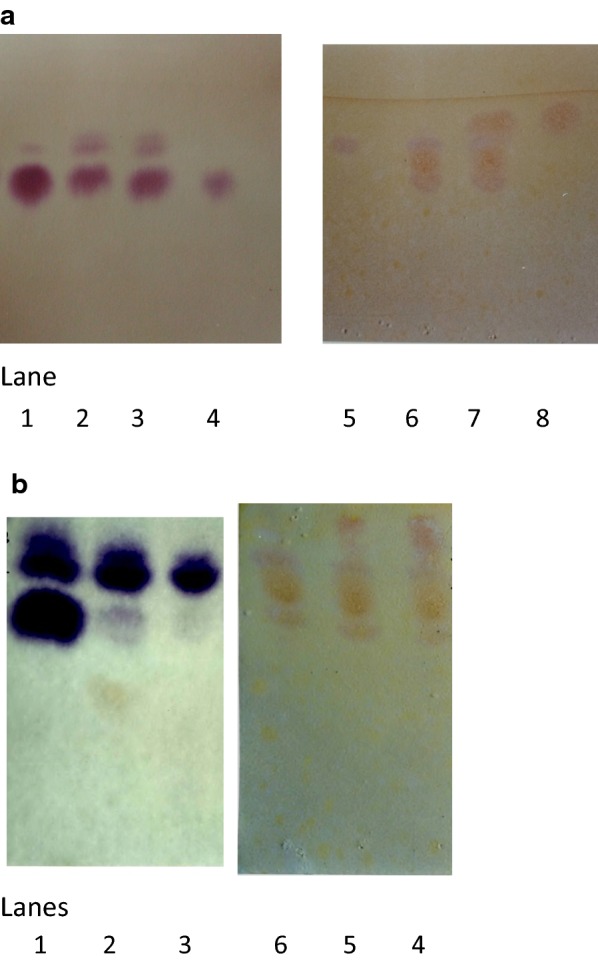
**a** TLC of polyamide waste. TLC plates showing components of solubilised oligomer waste before and after hydrolysis using acid (2 M sulphuric acid at 130 °C). The chromatogram was developed using the solvent system composed of *n*-propanol: ethyl acetate: ammonia: water (6:1:1:3) to resolve the linear components which were detected using ninhydrin reagent. Alternately the chromatogram was developed using the solvent system composed of *n*-butanol: acetic acid: water (12:3:5) to resolve the cyclic components which were detected using Dragendorff’s reagent. Lanes 1, 2, 3 show linear components after acid hydrolysis at 4.5 h, 0.5 h, 0 h. Lane 4: standard 6-ACA. 0 h is the unhydrolysed sample showing ACA, linear dimer, linear trimer; Rf 0.6, 0.81, 0.89 respectively. Lanes 5, 6, 7 show cyclic components after acid hydrolysis at 4.5 h, 0.5 h, 0 h. Lane 8: standard caprolactam. 0 h is the unhydrolysed sample showing caprolactam, cyclic dimer, cyclic trimer, cyclic tetramer, cyclic pentamer; Rf 0.79, 0.73, 0,68, 0.64, 0.56 respectively. **b** TLC of polyamide waste treated using bacteria*. *Solubilised oligomer waste was treated with a mixture of *Arthrobacter citreus, Rhodococcus rhodochrous*, *Bacillus sphaericus* and *Alcaligenes faecalis.* Lanes 1, 2, 3 showing linear components: 0 h, 24 h, 48 h after incubation; 48 h sample shows absence of 6-ACA. Lanes 4, 5, 6 showing cyclic components: 0 h, 24 h, 48 h after incubation; 48 h sample shows absence of caprolactam

The waste from a nylon-6 polymer production industrial plant formed due to incomplete polymerisation of the monomer was in the form of a soft solid mass at ambient temperature and was routinely dumped on land at the site. This polyamide waste was alkaline (pH 10 to 11) and when solubilized in synthetic medium at 80 °C (1% w/v) the pH was 8.5. Using thin layer chromatography, the solubilised polyamide waste was found to be a mixture of several components which were monomers and incompletely synthesized oligomers and detected using ninhydrin or Dragendorff reagent. The sensitivity of Ninhydrin reagent is more than that of Dragendorff reagent and thus ninhydrin-positive components appeared to be in higher amount. Caprolactam which is the raw material for nylon-6 was identified (Fig. 1a lane 7) by comparison with standard caprolactam (Fig. 1a lane 8 relative front, Rf value 0.79) and the other lactam group containing components detected as orange red spots using Dragendorff’s reagent were cyclic oligomers, dimer (Rf 0.73), trimer (Rf 0.68), tetramer (Rf 0.64) and pentamer (Rf 0.56). The oligomers resolved in positions relative to each other. This waste also contained non cyclic, linear components and these were detected by ninhydrin reagent. Standard 6-aminocaproic acid, ACA (Fig. 1a lane 4, Rf 0.6) was matched with 6-ACA in the waste (Fig. 1a lane 3) and the other components were linear dimer, Rf 0.81 and linear trimer, Rf 0.89. When synthetic medium containing such solubilised oligomer waste was inoculated with mixture of caprolactam-degrading bacteria, *A. citreus*, *R. rhodochrous*, *B. sphaericus* and *A. faecalis*, after incubation, 6-ACA was degraded and its oligomers were decreased (Fig. 1b lanes 2, 3) and caprolactam was also degraded (Fig. 1b lane 6). The medium with the solubilised waste initially contained 0.32% caprolactam and after 48 h it was found to be less than 0.03%. Thus biodegradation of caprolactam, the toxic component was achieved. There are no reports of toxicity of oligomers, but to study the possible fate of conversion of oligomers due to hydrolysis, the oligomer waste was subjected to acid hydrolysis using 2 M sulphuric acid at 130 °C for 0.5 to 5 h. Such acid-hydrolysed polyamide waste eventually showed only one major component: 6-ACA (Fig. 1a lanes 1, 5).

### Biodegradation of caprolactam using Gram-positive bacterial cultures

**Fig. 2:**
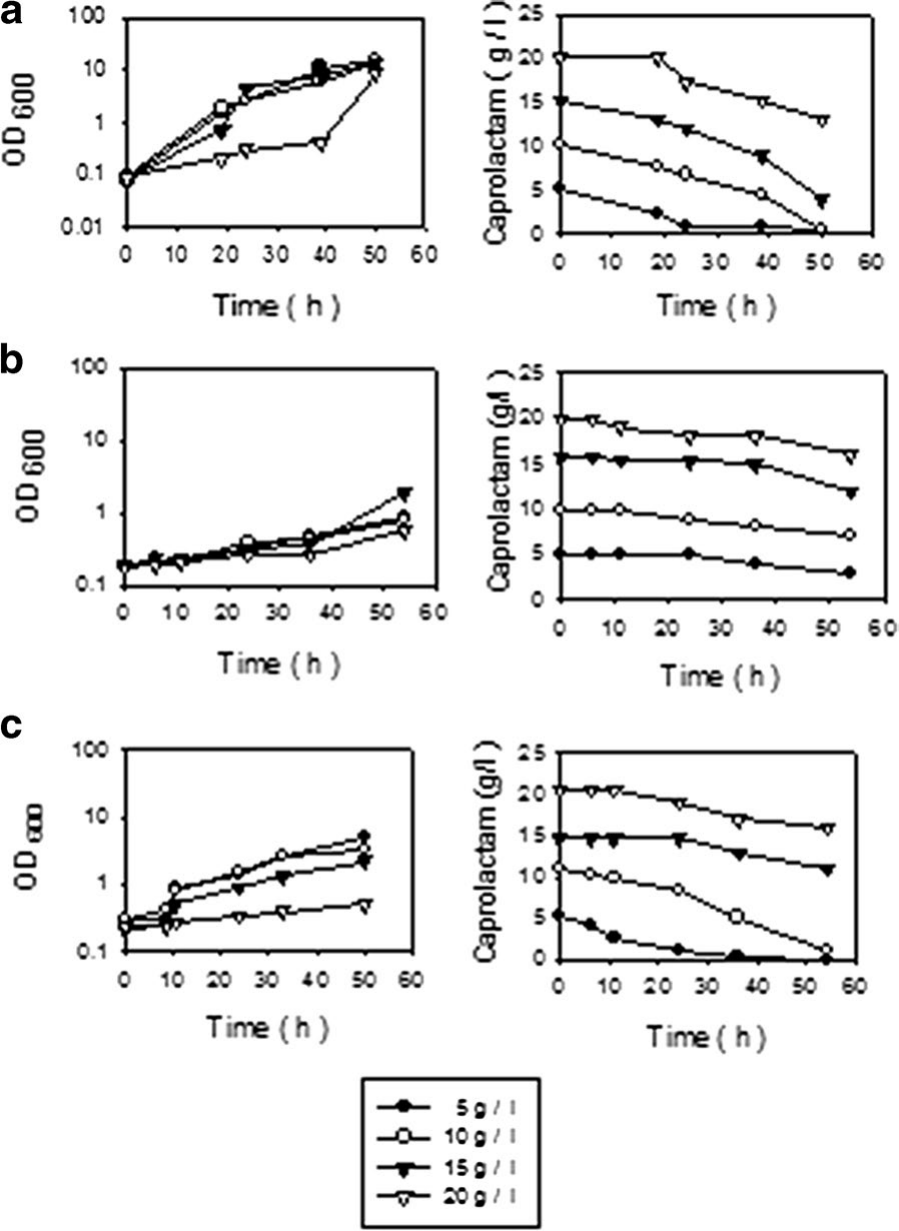
Degradation of caprolactam by Gram positive bacteria. Basal synthetic media contained caprolactam as the sole source of carbon (filled circle 5 g/l, open circle 10 g/l, filled inverted triangle 15 g/l, open inverted triangle 20 g/l). NH_4_Cl (1 g/l) was added as additional nitrogen source and flasks were inoculated with bacteria and incubated on a rotary shaker up to 60 h. **a** *Arthrobacter citreus*, **b** *Bacillus sphaericus*, **c** *Rhodococcus rhodochrous*

Amongst all the components of the oligomer waste, caprolactam is reported to be toxic to various forms of life. In situ biodegradation of caprolactam of the polyamide waste is required for the bioremediation of the soil contaminated by the waste dumped on land and for this a potent caprolactam-degrading bacterial culture is required. Bacterial strains which are proposed to be used for bioremediation of such toxic-waste contaminated soil or wastewater should have high specific caprolactam-degradation rate. Thus the individual Gram positive caprolactam-degrading soil bacteria *A. citreus*, *R. rhodochrous*, *B. sphaericus* were compared for their efficiency to utilise caprolactam. The synthetic medium used contained caprolactam as the only source of carbon for the bacteria. Growth of bacteria was found to increase proportionately to substrate utilization in logarithmic phase. At 0.5% caprolactam concentration where there was probably neither substrate excess nor substrate toxicity, *A. citreus* grew profusely and reached log phase in 24 h and degraded caprolactam efficiently and by 40 h culture reached stationary phase (Fig. 2 ). *A. citreus* had a maximum specific growth rate (μ) of 0.14, 0.19, 0.12 h^−1^ in medium containing 5, 10, 15 g/l respectively of caprolactam. *Bacillus sphaericus* was not able to bring about degradation of caprolactam efficiently and *R. rhodochrous* was able to degrade caprolactam but less efficiently as compared to *A. citreus*. Bacterial strains which are proposed to be used for bioremediation should also be tolerant to high (shock) dose of caprolactam because batches of waste may differ with respect to the concentration of caprolactam. *A. citreus* was found to degrade up to 20 g/l caprolactam and this isolate was selected for further studies. The minimum inhibitory concentration (MIC) of caprolactam for *A. citreus* was 2.25% to 3.5% in solid medium or broth containing caprolactam. The minimum bactericidal concentration (MBC) was determined by inoculating aliquots from the tubes showing no growth in presence of caprolactam into fresh medium without caprolactam and the MBC of caprolactam was 4%. Thus caprolactam up to 4% concentration did not bring about killing of the cells, rather the cells were only inhibited. There are no earlier reports of MBC of caprolactam for bacteria. But a comparison in case of structurally related beta lactam (4 membered) compound penicillin, known for its antibacterial effects and use as an antibiotic was done. MIC of penicillin for *A*. *citreus* was found to be 0.04 U/ml and MBC was 1 U/ml (Table 1). The growth ability of caprolactam degrading bacteria in presence of beta lactam compound such as penicillin has also not been reported yet.

**Table 1:** Effect of penicillin on growth of *Arthrobacter citreus*

Concentration of penicillin Unit/ml	Growth in presence of penicillin	Growth after transfer of cells to medium without penicillin
0	+	+
0.02	+	+
0.04^a^	−	+
0.1	−	+
0.2	−	+
0.4	−	+
0.8	−	+
1^b^	−	−

+: growth of cells (turbidity), −: no growth

^a^Minimum inhibitory concentration

^b^Minimum bactericidal concentration

### Accumulation of extracellular products during utilisation of caprolactam by *A*. *citreus*

**Fig. 3:**
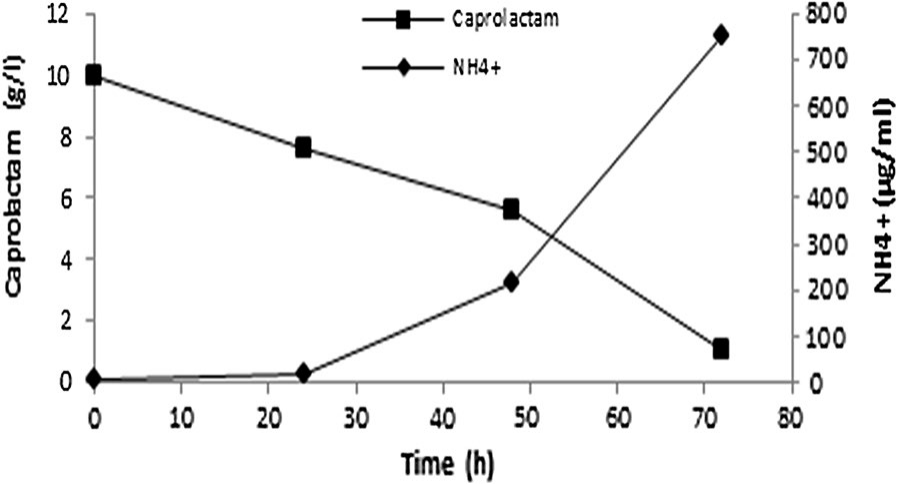
Formation of ammonium while utilisation of caprolactam* by *A*. *citreus*. *Basal synthetic medium containing 10 g caprolactam/l as the sole carbon and nitrogen source was inoculated with *A. citreus*

**Fig. 4:**
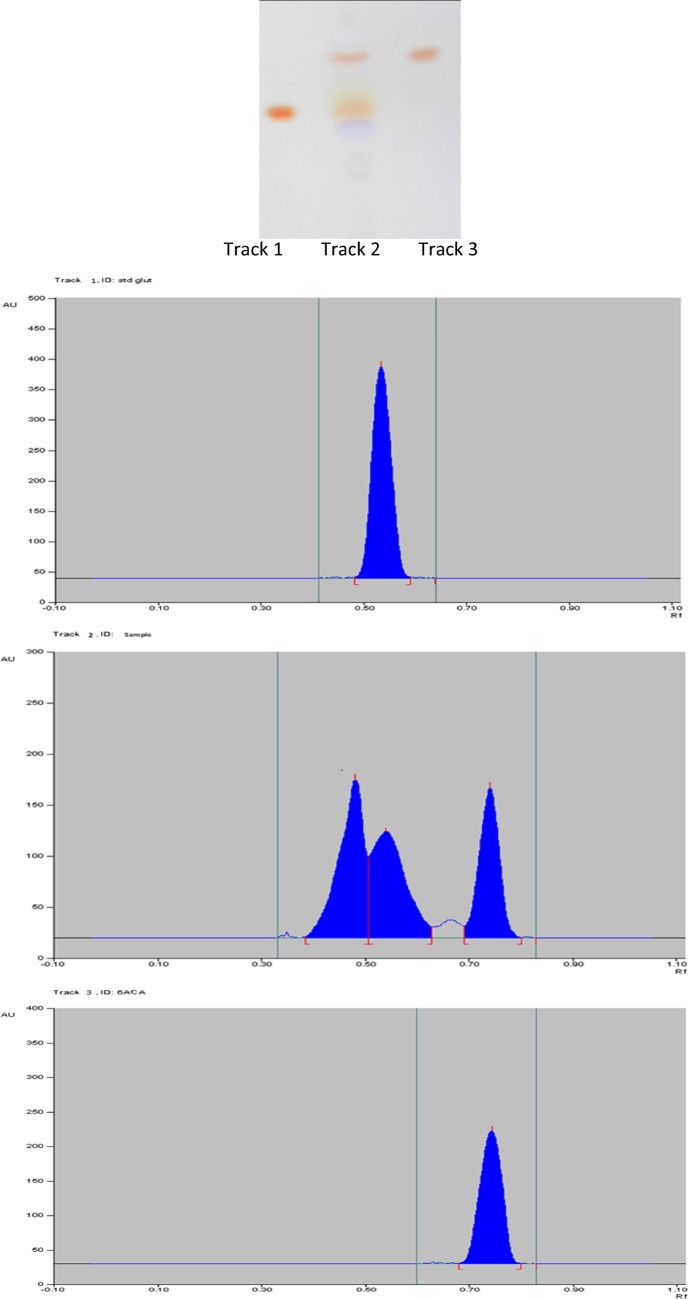
HPTLC of culture supernatant of *A. citreus* during utilisation of caprolactam. Detection of spots on chromatogram was done using ninhydrin reagent. After development of spots, plates were scanned for output of Rf and peak area (Table 2). 0 h sample/control medium (not shown) showed no spots. Loaded: Track 1: standard glutamic acid, Track 2: culture supernatant, Track 3: standard 6-ACA

For further studies using *A. citreus*, caprolactam was supplied at concentration of 1% in synthetic medium as sole carbon as well as sole nitrogen source *without any additional nutrients or growth factors*. Initially there was no ammonium salt in medium, and so the liberation of ammonia into extracellular medium during degradation of caprolactam by *A. citreus* (Fig. 3) indicated that the ammonium was formed from the nitrogen from caprolactam assimilated by cells. *A. citreus* was tolerant to the resulting high pH (8–9) due to the presence of high concentration of ammonium in medium as incubation proceeded. The first product obtained due to microbial degradation of ε-caprolactam is reported (Shama and Wase 1981) to be the corresponding linear compound, ε-aminocaproic acid (6-aminocaproic acid, 6-ACA) and after further metabolism the other products produced are adipate and succinate which enter into the tricarboxylic acid cycle. In our study also *A. citreus* was found to accumulate 6-ACA in the extracellular medium during degradation of caprolactam at some time intervals (Fig. 4 , Table 2). Interestingly, another amino acid-*glutamic acid* was also found in extracellular medium while degradation of caprolactam by *A. citreus*. Glutamic acid always accumulated in broth when either only caprolactam was present as sole carbon and nitrogen source and even when caprolactam along with sodium citrate (another carbon source) was present in medium and also in medium with caprolactam and low phosphate concentration where growth was decreased as compared to normal medium. So far, in literature it is not reported that bacteria accumulate glutamic acid during caprolactam degradation, though accumulation of 6-aminocaproic acid is usually reported. One more ninhydrin positive compound was detected but could not be identified. The selection of spots for identity and spot area in HPTLC was done by the inbuilt software. At zero hour there were no corresponding spots as compared to the sample after incubation with *A. citreus* (figure not shown). Using HPTLC, quantitation was done and 6-ACA and glutamic acid were estimated to accumulate 0.01% each (Fig. 4). Further, for confirmation, the presence of glutamic acid was ascertained and quantitated by a *spectrophotometric assay selective and specific* for glutamic acid which has been validated in literature for glutamic acid even if it is present in a protein sample along with all other amino acids. The other amino acids do not give this reaction and hence do not interfere in the estimation. In our study the non interference of other aminoacids in the estimation protocol was checked using cysteine as a representative amino acid. The maximum glutamic acid found accumulated in 48 h old growing culture supernatant of *A. citreus* while growing in medium with 1% caprolactam was 0.13%. In the same sample 6-ACA was found to be 0.1% using ninhydrin based spectrophotometric estimation. Caprolactam was thus degraded by *A. citreus* and non toxic amino acid products were formed as intermediates.

**Table 2:** Amino acids formed during utilisation of caprolactam by *A. citreus*

Track	Peak	Max position	Max height	Area	Assigned substance	Concentration μg/volume used
1	1	0.54 R_f_	350.5 AU	12,710.0 AU	Glutamic acid	6 μg/3 μl
2	1	0.48 R_f_	155.0 AU	7084.5 AU	Unknown	
2	2	0.54 R_f_	104.7 AU	6461.0 AU	Glutamic acid	3 μg/30 μl
2	3	0.75 R_f_	147.2 AU	5424.9 AU	6-ACA	2.2 μg/30 μl
3	1	0.75 R_f_	192.6 AU	7886.3 AU	6-ACA	3 μg/3 μl

HPTLC analysis of culture supernatant of *A. citreus* during utilisation of caprolactam was done. The developed plate (Fig. 4) was scanned for components and peak area. 0 h sample/control medium showed no spots

Track 1: glutamic acid; Track 2: sample; Track 3: 6-ACA

**Fig. 5:**
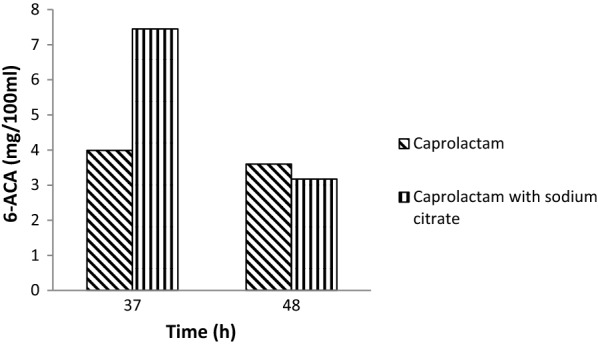
Accumulation of 6-aminocaproic acid during degradation of caprolactam by *A. citreus.* The media used contained either only caprolactam (10 g/l) or caprolactam with sodium citrate (1 g/l) and were inoculated with *A. citreus*

**Fig. 6:**
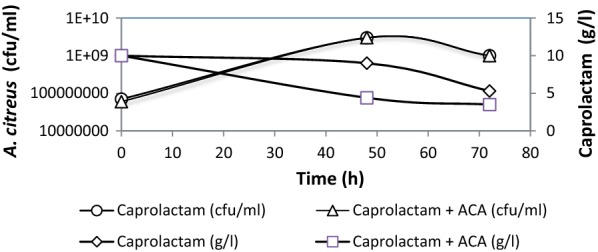
Utilisation of caprolactam by *A. citreus* in presence of 6-ACA. The media contained either only caprolactam (10 g/l) or caprolactam supplemented with 6-ACA (5 g/l) and were inoculated with *A. citreus*. Open circle, open triangle: cfu in medium with either only caprolactam or caprolactam supplemented with 6-ACA respectively. Open square, open diamond: residual caprolactam in medium with either only caprolactam or in medium supplemented with both—caprolactam and 6-ACA respectively

The formation of 6-ACA also occurred in presence of sodium citrate provided as additional easily utilizable carbon source along with caprolactam (Fig. 5). It is a known fact that presence of high amount of a major intermediate or any intermediate product of a catabolic pathway of a compound may bring about decrease in degradation of that compound. Thus to check whether the presence of 6-ACA affects the degradation of caprolactam when both are simultaneously present in polyamide waste, the degradation by *A. citreus* was studied in presence of additionally supplied 6-ACA in the usual medium containing caprolactam. It was found that *A*. *citreus* could degrade caprolactam even in presence of 6-ACA (Fig. 6) though there was a decrease in the rate of caprolactam utilization. The number of viable cells increased 100 fold when either only caprolactam or caprolactam with 6-ACA were provided for growth because both substrates are utilizable by *A. citreus*.

### Degradation of caprolactam by *A. citreus* in presence of low phosphate and in soil

**Fig. 7:**
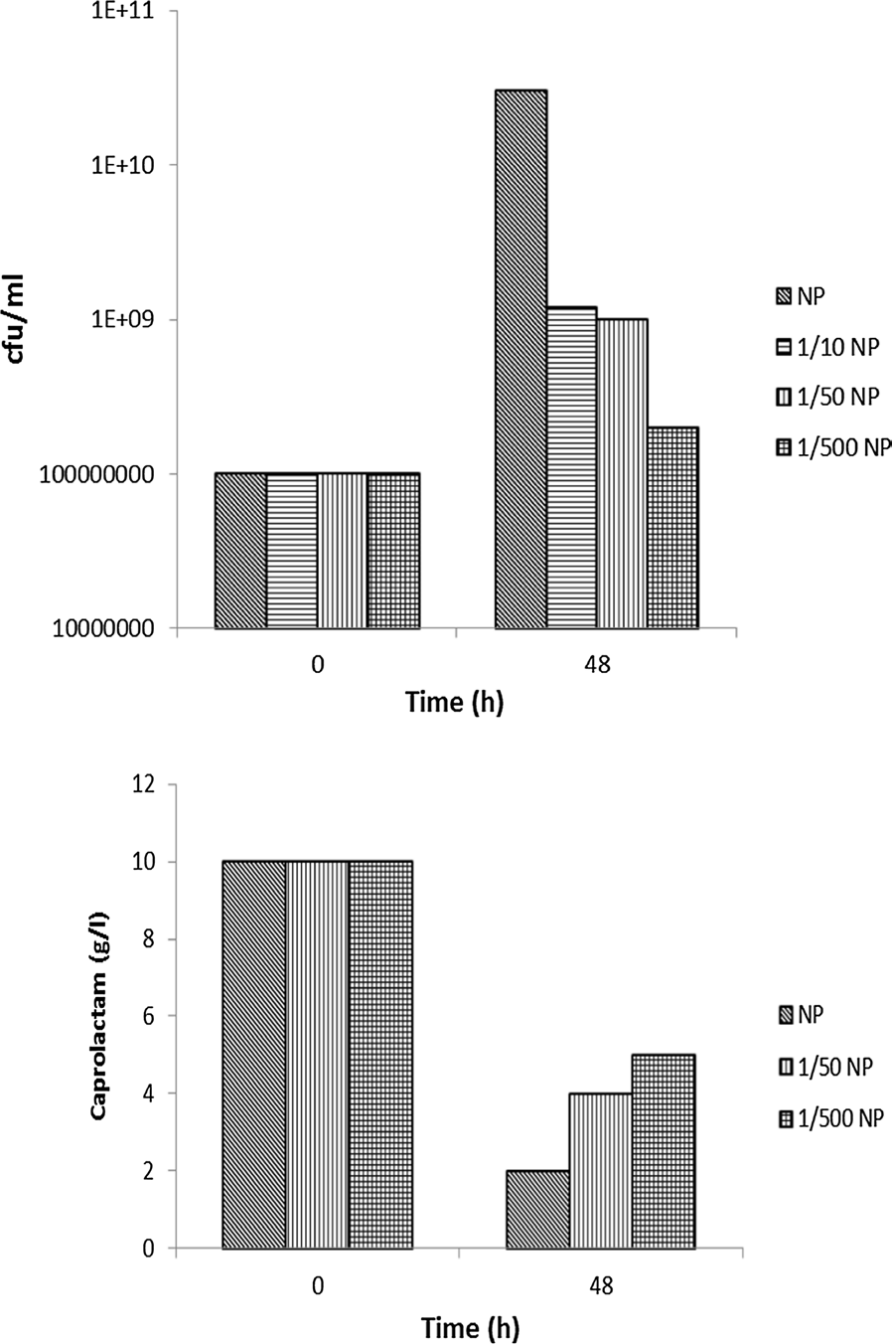
Utilisation of caprolactam by *A. citreus* in media with different phosphate concentration. The basal synthetic media contained caprolactam and inorganic phosphate salts to attain C:P of 100:3 (Normal P; NP), 100:0.3 (1/10 NP), 100:0.06 (1/50 NP), 100:0.006 (1/500NP)

**Fig. 8:**
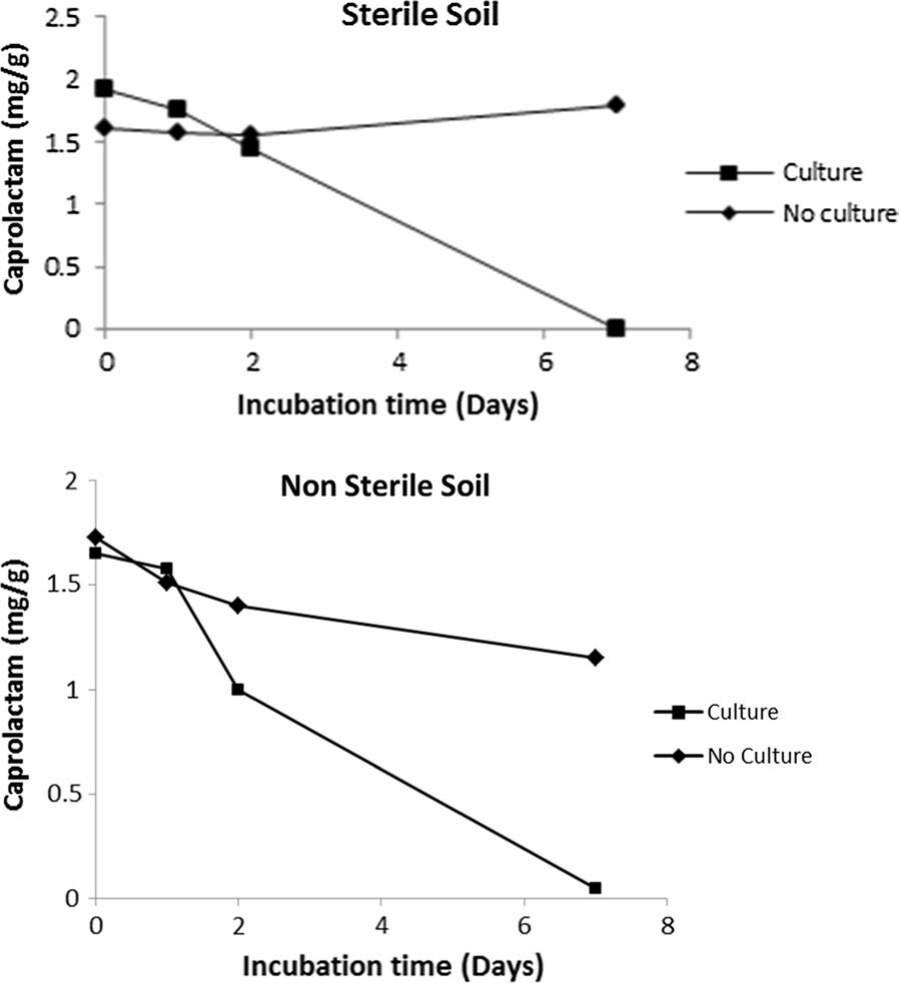
Degradation of caprolactam in soil by *A. citreus*. For the study of biodegradation of caprolactam in soil, fertile garden soil was used. 5 g of dry sterile soil (autoclaved twice) or fertile-non sterile soil (10^11^ cfu of natural soil flora/g dry soil) in 50 ml sterilised vials, was mixed with 10 mg caprolactam and a minimal amount of sterile water, inoculated (filled square) with *A. citreus* (10^8^ cfu/g soil) and incubated at 30 (± 2) °C for 7 days. Soil not inoculated with culture (filled diamond) was kept as control. By addition of sterile water the soil was kept sufficiently moist. At various time intervals, 5 ml of sterile water was added to the entire system, the contents were vigorously vortexed and the aqueous extract was centrifuged at 10,000×*g* for 20 min and the supernatant was used for estimation of caprolactam

While using synthetic medium with caprolactam as the sole source of carbon and nitrogen for the bacteria, the C:N ratio due to presence of caprolactam (C_6_H_11_NO) will be 100:15 and irrespective of any concentration of caprolactam this ratio will be a constant. In the soil contaminated by the dumped oligomer waste the carbon and nitrogen supply for the inoculated bacteria used for biodegradation will be contributed by caprolactam and other waste components. In literature it is reported that additional carbon maybe required to be provided to meet the recommended optimum C:N value of 100:10 for bacterial growth (Esikova et al. 2012). This approach may be feasible in laboratory media or even in wastewater treated in treatment plant reactors but in situ in soil this is not feasible as it will further add to organic load. Thus in our study additional carbon was not added. Similarly a C:P ratio of 100:1 is required for bacterial growth but in situ soil nutrients are in a limiting concentration. In order to check the survival and suitability of *A. citreus* to be able to degrade caprolactam in such conditions, medium containing only caprolactam as source of carbon and nitrogen with 0.6 g K_2_HPO_4_ and 0.2 g KH_2_PO_4_/l (C:P ratio of 100:3) or medium with caprolactam and lower concentrations of phosphates to attain lower C:P ratios of 100:0.3, 100:0.06, 100:0.006 was used. Even in low phosphate condition *A. citreus* was able to show increase in growth (viable count of cells) and degrade caprolactam, although to a lesser extent than in case where there was sufficient phosphate (Fig. 7). In order to assess whether *A. citreus* was suitable for treatment of the caprolactam in soil, soil with caprolactam was inoculated with *A. citreus* and caprolactam was the sole carbon and nitrogen source added. The soil used was either sterile soil (autoclaved twice) or non sterile (10^11^ cfu/g dry soil). Caprolactam was found to be degraded at end of 7 days (Fig. 8). Thus the *A. citreus* strain used in our study can be used for degrading caprolactam present in oligomer waste which is disposed on land near nylon-6 polymer production plants.

## Discussion

The aim of chemical industries is to achieve maximum conversion of raw materials into product. However in case of production of the polymer nylon-6, some amount of oligomer waste is produced unintentionally due to incomplete polymerisation of monomer caprolactam. The separation and identification of such incompletely synthesised oligomer components of the solid waste by TLC showed a total of eight (three linear and five cyclic) components (Fig. 1a), depending on polarity and pH of the solvent system used to develop the chromatogram, and molecular weight, polar or hydrophobic nature, water solubility of components and different detection reagents used. In literature only the Rf of components of pure monomers or, individual oligomers specifically synthesised has been reported (Kobayashi 1966; Negoro et al. 2012). The Rf of components of the oligomer waste mixture was thus compared with these values but the exact values could not be considered, rather the relative positions were used to identify the components present in the waste and detected by specific reagents (Fig. 1a). Chemical (acid) hydrolysis of the oligomer waste showed that hydrolytic conversion of all cyclic and linear oligomers lead to increase in amount of one component: 6-ACA (Fig. 1a). The polyamide waste contained only one toxic component initially: caprolactam. It was found to be degraded by treatment with bacteria in 48 h thus making the waste free of the toxic component (Fig. 1b). In earlier reports, degradation was studied in media (Fukumura and Teramura 1982; Esikova et al. 2012; Esikova and Taran 2016) where caprolactam was not in present along with other oligomers or monomers all of which can influence degradation in a mixed substrate. Our present studies showed biodegradation of caprolactam in oligomer waste mixture which was initially in form of solid waste, with alkaline pH which is unfavourable for microbial activity.

According to literature, most of the caprolactam-degrading bacteria reported are Gram negative for example *Pseudomonas* spp., *Proteus* sp., *A. faecalis* and others (Boronin et al. 1986; Otzen et al. 2018; Sanuth et al. 2013; Baxi and Shah 2002) and few Gram positive caprolactam-degrading bacteria have been reported as compared to Gram negative bacteria. As compared to Gram negative bacteria, the more predominant members found in soil are the Gram positive bacteria (Maier and Pepper 2000) and such bacteria are also more inherently resistant to stress in environment (Schimel et al. 2007; Mongodin et al. 2006). In the present work, the aim was to bring about in situ bioremediation of soil contaminated with caprolactam-containing waste and thus the individual caprolactam-degrading Gram positive soil bacteria *A. citreus*, *R. rhodochrous*, *B. sphaericus* were compared for potential to degrade caprolactam. *A. citreus* was the most potent amongst the three cultures with μmax 0.19 h^−1^ at 1.5% caprolactam substrate (Fig. 2) during the logarithmic phase of growth. The same final OD_600_ attained at various concentration of caprolactam after logarithmic phase in case of *A. citreus* indicated that cells entered stationary phase in after 24 up to 48 h whilst the other cultures were still in early or mid log phase. Thus *A. citreus* can be used as a single potent culture for degradation of caprolactam in wastes. In a recent report (Otzen et al. 2018), the caprolactam-utilising bacterium *Pseudomonas jessenii* GO3 which was studied extensively for enzymes and the genes coding for the enzymes of caprolactam degradation pathway was reported to grow with caprolactam up to maximum of only 0.46% and μmax at this concentration was less than 0.01 h^−1^. The MIC of caprolactam for *A. citreus* was 2.25% to 3.5% in solid medium or broth but the MBC value was 4% as expected because MBC values are usually higher than MIC values (Valle et al. 2016) and the MBC value for *A. citreus* is the highest as compared to all caprolactam-degrading bacterial isolates reported so far including Gram positive isolates *Corynebacterium*, *Bacillus cereus* and *Gulosibacter* sp. (Shama and Wase 1981; Mehta et al. 2014; Esikova and Taran 2016). Caprolactam has an epsilon lactam ring which is a five membered ring and being a lactam compound it is likely to have effect on cell wall synthesis of bacteria. Similar ring structures are found in beta lactam compounds such as penicillin which have a four membered ring, and these compounds have anti-bacterial properties. There are no earlier reports of caprolactam degrading bacteria in relation to beta lactam compounds. A comparison was made with the concentration of penicillin in the commercially available antibiotic discs used for checking of antibiotic susceptibility of bacteria. These disks contain 1 U/ml and 10 U/ml of penicillin for Gram positive and Gram negative bacterial cultures respectively. *A citreus* thus can be considered to be intermediate or tolerant because MBC of penicillin for *A. citreus* was 1 U/ml (Table 1).

Caprolactam is a xenobiotic compound but it was used as sole carbon and energy and as well as sole nitrogen source. Thus caprolactam provided at 1% was converted to several products some of which were detected in extracellular medium. Initially no inorganic nitrogen source such as ammonium salts was provided in the medium and so the detection of ammonia in the culture broth during degradation of caprolactam by *A. citreus* (Fig. 3) was an evidence that the nitrogen of caprolactam was also assimilated and converted to ammonia. Amongst the other products detected in culture broth, ε-aminocaproic acid (6-ACA) was found to accumulate extracellularly (Fig. 4) as also in the case of earlier reports of caprolactam degradation pathway (Shama and Wase 1981; Otzen et al. 2018). This implied that the *A. citreus* soil strain was bringing about degradation of caprolactam via the already characterised pathway of caprolactam degradation and this is important especially in environmental conditions where it is required to prove that intermediates are not more toxic than parent xenobiotic compound. 6-ACA was found to accumulate and so in order to check whether presence of 6-ACA can adversely affect degradation of caprolactam by *A. citreus*, it was supplied externally in high concentration, but was not found to inhibit the degradation of caprolactam (Figs. 5 and 6). Although a decrease in caprolactam utilization was seen when two substrates—caprolactam and ACA were provided to the culture, growth attained was identical in both cases In earlier reports the growth of some *Pseudomonas* cultures was reported to be affected by the accumulated 6-ACA during degradation of caprolactam (Litvinenko et al. 1993).

Our study *for the first time* describes the finding of extracellular *accumulation of glutamic acid by a caprolactam*-*degrading bacterial strain*, *A. citreus* (Fig. 4, Table 2). The products reported to be formed after 6-ACA are adipic acid and succinic acid, which enter into the tricarboxylic acid cycle and the main enzymes of the caprolactam-degradation pathway are ATP dependent caprolactam hydrolase and 6-ACA transaminase (Shama and Wase 1981; Otzen et al. 2018). So far, in literature it is not reported that bacteria accumulate glutamic acid in extracellular medium during caprolactam utilisation especially when caprolactam is supplied as sole source of carbon and nitrogen. Glutamic acid an important amino acid involved in metabolism of nitrogen containing compounds in a cell. Glutamic acid also has several food, pharmaceutical and other applications. Glutamic acid is one of the amino acids produced commercially by bacterial fermentation using strains of *Corynebacterium glutamicum* and using high concentration of nutritionally optimized carbohydrate such as glucose from starch, or molasses or even from acetic acid and ethanol but with a high concentration of a nitrogen source in form of ammonia, inorganic N salt, or urea (Kumagai 2006). In our report the substrate utilized is a *xenobiotic* compound-caprolactam, *used as sole source of carbon and nitrogen* and the accumulation of glutamic acid in extracellular medium probably occurs along with high ammonium concentration prevalent. Further, most glutamic acid-producing strains reported are Gram positive non sporulating, nonmotile, cocci or rod like bacteria in which the 2-oxoglutarate formed in the tricarboxylic acid cycle is aminated to glutamate by the action of glutamate dehydrogenase. Members of the genus *Arthrobacter* are related to corynebacteria and thus probably the *A. citreus* wild type strain used in the present study has the potential to accumulate glutamic acid. This wild type *A. citreus* strain can be further studied for conversion of native oligomer waste containing caprolactam into glutamic acid or 6-ACA. 6-ACA is well known as an anti-fibrinolytic drug (Nilsson 1980). Thus both the amino acids produced by *A. citreus* during caprolactam degradation: 6-ACA and glutamic acid have important applications.

For optimal growth and degradation of caprolactam by bacteria it is mentioned in literature that additional carbon is required to be provided to meet the required C:N:P ratio of 100:10:1 (Esikova et al. 2012) but in soil in situ such addition is not feasible and will further add to organic pollution of the soil ecosystem. For in situ bioremediation technology to be successful, inorganic nutrients should also be available sufficiently for bacteria However, *A. citreus* was able to degrade caprolactam (C:N 100:15) without additional organic nutrients in soil and even in presence of low phosphorous (Figs. 7 and 8). The isolate *A. citreus* was a soil isolate and several *Arthrobacter* species are reported as dominant heterotrophic bacteria which are highly aerobic, nutritionally non exacting and nutritionally versatile and thus reported to be suitable for biodegradation of glyphosate, methyl tert-butyl ether, 2,4-dichlorophenoxyacetate, nitroglycerine and several other compounds (Mongodin et al. 2006). *Arthrobacter* strains found in soil have inherent capacity to survive even for long period in harsh environment induced because of starvation, ionizing radiation, oxygen radicals and toxic chemicals. All the results of the present study prove the suitability of *A. citreus* as a potentially promising bacterium for in situ bioremediation of soil contaminated with caprolactam. In conclusion the caprolactam-degrading strain *A. citreus* is a promising strain having potential in the fields of environmental microbiology and microbial technology.

## Data Availability

At MTCC, IMTECH, India.
